# Supplementary material for: Autosomal allelic inactivation at loci with variable replication timing and dosage sensitivity
Source: eLife. 2026 Jul 8;15:RP109938. doi: 10.7554/eLife.109938 (PMC13345637; doi:10.7554/eLife.109938)
Supplement: Peer review history

# Editor report: eLife Assessment

**DOI:** 10.7554/eLife.109938.4.sa0

**Authors:** Adèle L Marston

**Affiliations:** University of Edinburgh United Kingdom

**Keywords:** Important, Solid

This **important** study links allelic expression imbalance with replication timing, suggesting a stochastic model for haploinsufficiency in dosage-sensitive disease. The integration of allele-specific RNA-seq and replication timing in clonal systems provides **solid** evidence for an association between asynchronous replication and allelic imbalance, although the scope and generality should be addressed in future work. This study will interest epigeneticists and genome regulation researchers studying replication timing and monoallelic expression, as well as developmental biologists and human geneticists concerned with clonal heterogeneity, haploinsufficiency, and variable disease penetrance.

[Editors' note: this paper was reviewed by Review Commons.]

# Referee report: Reviewer #2 (Public review)

**DOI:** 10.7554/eLife.109938.4.sa1

[Editors' note: this version has been assessed by the Reviewing Editor without further input from the original reviewers. The authors have addressed the comments raised in the previous round of review.]

The authors pair analysis of replication timing and allele-specific expression in clonal populations of primary human cells. They combine these data with previously published data on clones from transformed human cell lines. They identify a number of genomic regions that display asynchronous replication timing in at least one clone and correlate these regions with allele-specific expression of genes within them. They also observe that several interesting gene sets, including genes that are associated with human diseases, map to asynchronously replicating regions. This is a good experimental approach that builds on already published data demonstrating the connection between allelic imbalance and replication timing.

# Author comment: Author response

**DOI:** 10.7554/eLife.109938.4.sa2

**Authors:** Michael B Heskett, Athanasios E Vouzas, Brian Johnstone, Krister P Freese, Phillip A Yates, Philip F Copenhaver, Paul T Spellman, David M Gilbert, Mathew J Thayer

**Affiliations:** David Geffen School of Medicine at UCLA Los Angeles United States; San Diego Biomedical Research Institute San Diego United States; Oregon Health and Science University Oregon United States; Department of Orthopedics and Rehabilitation, Oregon Health and Science University Portland, Oregon United States; Oregon Health and Science University Oregon United States; Oregon Health and Science University Oregon United States; University of California, Los Angeles Los Angeles United States; San Diego Biomedical Research Institute San Diego United States; Department of Chemical Physiology and Biochemistry, Oregon Health and Science University Oregon United States

The following is the authors’ response to the previous reviews

**Reviewer #2 (Public review):**

Summary:

The authors pair analysis of replication timing and allele-specific expression in clonal populations of primary human cells. They combine these data with previously published data on clones from transformed human cell lines. They identify a number of genomic regions that display asynchronous replication timing in at least one clone and correlate these regions with allele-specific expression of genes within them. They also observe that several interesting gene sets, including genes that are associated with human diseases, map to asynchronously replicating regions. This is a good experimental approach that builds on already published data demonstrating the connection between allelic imbalance and replication timing.

- This is a research topic that touches on a few sub-fields of biology, and thus to make the paper more approachable we would recommend a careful edit of the text for clarity and precision of language.

We thank the reviewers for their thoughtful and constructive comments, which substantially improved our manuscript. In response, we have revised the text and figures throughout to address the points raised.

- Authors point out that this is a decades-old field; we would suggest to use terminology established within the field is possible. Allelic imbalance has been referred to as AI, MAE (monoallelic expression), RMAE (random monoallelic expression) etc. The paper whose mouse data the authors make use of uses Asynchronous Stochastic Replication Timing (ASRT) instead of VERT to refer to the same phenomenon.

While we agree that allelic expression imbalance has been described by different investigators using many different phrases, we believe that MAE, RMAE and AI do not represent accurate descriptions of the phenomenon. We point out that “Allelic Expression Imbalance” has been used to describe this variable allelic expression by other investigators >120 times in the Pubmed database. In our study [and our previous study; Nat Commun. 2022; 13(1):6301] we used clonal analysis of allele-specific expression and found that while some clones display equivalent levels of expression between alleles of a given gene (i.e. bi-allelic expression) other clones express only one allele (i.e. mono-allelic expression), and yet other clones have undetectable expression (i.e. silent on both alleles). This pattern of allele-restricted expression indicates that each allele independently adopts either an expressed or silent state. Importantly, because these expression states are mitotically stable, allele-autonomous, and independent of parental origin, we refer to the choice of the expressed allele as stochastic. Given this variability, we believe that the phrase “Allelic Expression Imbalance” (AEI) represents a more accurate descriptor for this phenomenon.

In addition, the replication asynchrony that exists at these loci is not consistent with purely ASynchronous Replication Timing (ASRT) between alleles. We found that each allele can independently adopt either earlier or later replication timing in different clones. This variability results in some clones exhibiting pronounced asynchrony between alleles, while in others, the two alleles replicate synchronously, with both adopting either the earlier or later timing state. As reported in our previous study (Nat. Commun. 2022; 13:6301), this behavior reflects a stochastic and allele-autonomous process, leading us to describe these loci as exhibiting Variable Epigenetic Replication Timing (VERT), which we believe is a more accurate descriptor of this phenomenon.

- Methods do not provide fully sufficient detail to fully evaluate or reproduce these experiments.

We now provide a more detailed description of how VERT regions were identified, annotated, and quantified, including thresholds for allelic imbalance, replication timing variability, and sampling depth. We also justify the ≥80% AEI cutoff, which is based on recently published studies showing that modest allelic biases can have biological and clinical significance (Nature 2025; 637, 1186-1197). We also refer the readers to our recent description of these methods (Nat. Commun. 2022; 13:6301).

- It is helpful to show representative loci as the authors do in Fig 1F and G and Fig 2 but these panels are very densely rendered and thus difficult to process visually - even the cartoon version (1D) is thick with overlapping lines. The point that allelic imbalance is enriched in VERTs would be enhanced if the authors could present the allelic ratio for all genes found in all VERTs, demonstrating how replication timing on either chromosome affects the allelic ratio.

The stochastic nature of the allelic expression and replication timing observed at I/SCs is best visualized with each allele and each transcription unit displayed from multiple clones in the same panel. One of the goals of these figure panels is to emphasize that each I/SC has multiple transcription units that acquire expressed or silent states independently in each clone. Therefore, the expressed or silent status of one allele of a transcription unit does not predict expression status of the same or opposite allele of any other transcription unit within the same VERT region. In addition, the Early/Late pattern of replication timing that we detect is not correlated with which allele is transcriptionally active (see below). In these figure panels, we display each clone using different colors, each allele as solid or dotted lines, and each transcription unit based on chromosome position. While this arrangement makes for busy images, we believe that this format captures the full breadth of the variability in expression and replication timing that occurs at I/SCs.

Regardless, because each transcription unit is independent, we now provide the expression ratios for all transcripts that are generated from the VERT regions for the coding and non-coding transcription units in Figures 1, 2, and 6; shown in Supplemental Table 9. This analysis indicated that 4,017 informative reads were derived from the earlier replicating allele and 3,161 informative reads were derived from the later replicating allele, generating an allelic ratio of 1.3 (early/late) and a binomial P value of 1.0.

In addition, a similar analysis of imprinted loci revealed that even at genomic regions with parent-of-origin–specific expression, the replication timing of each allele does not align with transcriptional activity, i.e. both early- and late-replicating alleles can be transcriptionally active, depending on the gene. This observation is consistent with the complex organization of many imprinted domains, where genes on opposite alleles exhibit reciprocal expression patterns. To illustrate this point, we now include Supplemental Figure 1 demonstrating that imprinted loci harbor genes expressed from both the earlier- and later-replicating alleles. In addition, quantification of the total number of informative transcripts at the DLK1/MEG8 imprinted locus (Supplemental Figure 1a-1c) indicates that the ratio of transcripts derived from the early versus late replicating alleles is equivalent (i.e. an allelic expression ratio of 1.0; See Supplemental Table 9).

- The authors make the important point that VERTs are unlikely to be shared among different cell types and tissues (Fig 1i), but then find an enrichment for neuronal and immune genes in VERT regions identified in ACPs. It follows that these same genes are unlikely to be in such regions in the tissues where they are relevant. Some of the GO terms presented are too broad to suggest any biological significance to the result, even if there is statistical significance (for example, the top term for LCL clones 'Cytoplasm' is associated with 12,000 genes, and the second term for mouse clones 'Membrane' is associated with 10,000). It would be helpful to focus on GO terms lower in the GO hierarchy.

We now include our complete Gene Ontology analysis, with more specific biological categories, in Supplemental Table 5.

- Figure 3 highlights the association of related gene clusters with VERTs but the VERTs are assigned based on variable replication timing in just 1 or 2 clones. This is an interesting observation, but to make the point that "VERT regions frequently coincide with gene clusters in the human genome" there needs to be a systematic assessment of replication timing at all gene clusters across all clones, and a statistical test for significance.

Our intent in Figure 3 was not to suggest that all gene clusters are subject to VERT and AEI, but rather to highlight that several well-characterized multigene families that are known to exhibit AEI, such as olfactory receptor, protocadherin, and HLA gene clusters, coincide with VERT regions at their genomic locations. These examples serve as representative illustrations demonstrating that I/SC-associated regulation occurs at established AEI loci organized in gene clusters.

To clarify this point, we have revised the text to explicitly state that Figure 3 presents illustrative examples of known AEI-associated gene clusters overlapping with VERT regions, rather than a comprehensive or statistically exhaustive analysis of all gene clusters across the genome.

- It is an interesting hypothesis that VERTs are conserved between species at syntenic loci. If such regions are really conserved, one would expect that replication timing at these sites would be consistently asynchronous. However the data presented shows that in human clones these VERTs can be specific to an individual donor (as in 5A) or an individual clone (as in 5H).

As discussed in our Limitations Section, our analysis was restricted to a limited number of cell types, individuals, and clones, which may not capture the full diversity of I/SC usage across tissues and populations. While our dataset was sufficient to identify robust patterns of AEI and VERT, it likely represents only a subset of the broader landscape of I/SC regulation in both humans and mice. We anticipate that future studies incorporating a wider range of tissues, individuals, and clones will uncover an even greater degree of conservation and diversity in I/SC usage across genomes.

- The finding that VERTs coincide with neurodevelopmental disease genes in immune and cartilage cells is at odds with the previous statements and data about the tissue specificity of VERTs. In order to support the claim that neurodevelopmental disease associated genes reside in asynchronously replicating regions, and are thus more prone to allelic imbalance, it would be helpful if the authors demonstrated this phenomenon in neuronal cells.

We make two points that address this critique: First, many of the neurodevelopmental disease genes associated with VERT regions are not exclusively expressed in neuronal cells and have previously been shown to exhibit AEI in non-neuronal contexts. For example, Gimelbrant and Chess (Science, 2007; 318:1136–1140) demonstrated AEI of the Parkinson disease genes SNCA and LRRK2 in lymphoblastoid cell lines (LCLs), and in our previous study, that also used LCL cells, we detected AEI of DNAJC6, which is another Parkinson disease gene (Nat. Commun. 2022; 13:6301). In the present study, using cartilage progenitor cells, we identified VERT and AEI of several epilepsy-associated genes, including SCN1A, SCN2A (Fig. 6b), GABRA1(Fig. 6e), and SAMD12 (Fig. 6j), as well as a gene implicated in autism and neurodevelopmental disorders, SEMA5A (Fig. 5c), indicating that expression of these genes is not exclusive to neuronal cell types.

Second, independent studies from the Dr. E. Heard laboratory have provided further evidence that AEI occurs in neuronal lineages. Using mouse neural progenitor cells (NPCs), they identified genes subject to AEI (Dev. Cell, 2014; 28:366–380) and they later evaluated AEI of syntenic human neurodevelopmental disease genes, including Snca, App, Eya4, and Grik2 (Nat. Commun. 2021; 12:5330). In our data, we find that these mouse genes are located within VERT regions. In addition, and consistent with our use of AEI, they used the phrase “Allelic Expression Imbalance” to describe the epigenetic expression biases at these genes.

Together, these findings reinforce that AEI, and by extension I/SC regulation, is not restricted to specific cell types, but rather represents a generalizable mechanism of stochastic epigenetic regulation that includes genes relevant to neuro development and disease.

- The authors consistently lean on sparse samples (i.e. a single clone) within a modestly sized dataset (4 clones from 2 donors each) to propose a new model for haploinsufficiency in human disease. It may well be but the consistent focus on limited elements in the data and perhaps an overreach in the interpretation makes it difficult to appreciate the very good experiments presented.

We agree that our analysis was conducted on a modest number of cell types, individuals, and clones, which we explicitly acknowledge as a limitation of the present study. However, several key points support the robustness and broader relevance of our conclusions:

i) Clonal Design and Replication: The strength of our approach lies in its clonal resolution. Each clone represents a single-cell–derived population expanded to over a million cells, enabling direct detection of stable, mitotically heritable allele-specific epigenetic states that would not be apparent in population-averaged data. Importantly, many of the VERT regions we identified are shared between independent clones from different donors and across distinct cell types (ACP and LCL), demonstrating reproducibility and biological consistency.

ii) Cross-Species Validation: We further identified syntenic VERT regions in mouse pre-B cell clones, including at loci known to exhibit AEI in prior studies, providing independent validation and evolutionary conservation of the phenomenon.

iii) Integration with Published Evidence: Our findings extend prior observations of AEI and VERT (e.g. Gimelbrant et al. Science 2007; Heskett et al. Nat. Commun. 2022) and are fully consistent with known stochastic allelic expression imbalance of autosomal genes.

iv) We also draw parallels with the absence of cellular selection mechanisms that dictate dominant inheritance patterns for loss of function alleles for X linked disease genes (reviewed in: J Clin Invest, 2008, 20-23; and Nat Rev Genet. 2025, 26, 571–580). Our proposed model linking I/SC regulation to haploinsufficiency is therefore a synthesis of our results with an extensive body of published data, not an inference drawn from isolated observations.

v) Scope and Framing: We have revised the manuscript to clarify that our proposed model represents a mechanistic framework, not a definitive or exclusive explanation, for how stochastic allelic regulation could contribute to dosage-sensitive disease phenotypes. We also explicitly discuss the need for larger datasets and additional tissues to refine and test this model.

- This section refers to the revised version of the paper. We would like to thank the authors for the changes and explanations offered. Although we don't fully agree with a few answers offered, overall the answers and changes in the manuscript have significantly improved the work presented. As such it should be of interest to many readers.

We thank the reviewers for their thoughtful evaluation and constructive feedback. We appreciate their recognition that the revisions have strengthened the manuscript and are pleased that they find the work to be of broad interest.
